# Phosphatidylserine recognition and Rac1 activation are required for Müller glia proliferation, gliosis and phagocytosis after retinal injury

**DOI:** 10.1038/s41598-020-58424-6

**Published:** 2020-01-30

**Authors:** Kaori Nomura-Komoike, Fuminori Saitoh, Hiroki Fujieda

**Affiliations:** Department of Anatomy, School of Medicine, Tokyo Women’s Medical University, Tokyo, Japan

**Keywords:** Neuroscience, Anatomy

## Abstract

Müller glia, the principal glial cell type in the retina, have the potential to reenter the cell cycle after retinal injury. In mammals, proliferation of Müller glia is followed by gliosis, but not regeneration of neurons. Retinal injury is also accompanied by phagocytic removal of degenerated cells. We here investigated the possibility that proliferation and gliosis of Müller glia and phagocytosis of degenerated cells may be regulated by the same molecular pathways. After N-methyl-N–nitrosourea-induced retinal injury, degenerated photoreceptors were eliminated prior to the infiltration of microglia/macrophages into the outer nuclear layer, almost in parallel with cell cycle reentry of Müller glia. Inhibition of microglia/macrophage activation with minocycline did not affect the photoreceptor clearance. Accumulation of lysosomes and rhodopsin-positive photoreceptor debris within the cytoplasm of Müller glia indicated that Müller glia phagocytosed most photoreceptor debris. Pharmacological inhibition of phosphatidylserine and Rac1, key regulators of the phagocytic pathway, prevented cell cycle reentry, migration, upregulation of glial fibrillary acidic protein, and phagocytic activity of Müller glia. These data provide evidence that phosphatidylserine and Rac1 may contribute to the crosstalk between different signaling pathways activated in Müller glia after injury.

## Introduction

Müller glia, the principal glial cells in the retina, possess a variety of functions to support retinal neurons and act to maintain retinal homeostasis under physiological as well as pathological conditions^1,2^. In lower vertebrates like fish, retinal injury induces Müller glia to proliferate and dedifferentiate to neuronal progenitor cells that are capable of regenerating retinal neurons^3,4^. In mammals, however, such regenerative capacity of Müller glia is extremely limited. In rats, for example, Müller glia proliferate in response to injury, but they quickly exit the cell cycle and many undergo cell death possibly by DNA damage response^5^. In addition, Müller glia in mammals show a set of injury-induced responses called reactive gliosis, including cellular hypertrophy, migration, and upregulation of intermediate filaments such as glial fibrillary acidic protein (GFAP) and vimentin^6^. Although gliosis may be neuroprotective, it may hamper tissue repair if the reaction is prolonged^6^.

Previous evidence has indicated that the injury-induced responses of Müller glia are mediated by growth factors or cytokines secreted from damaged neurons, microglia, or Müller glia themselves^6–8^. A pioneering study by Rattner and Nathans^9^ showed that damaged photoreceptors produce endothelin2 (Edn2), which signals onto Müller glia and induces their reactive responses such as GFAP upregulation. However, the Edn2-mediated interaction between damaged photoreceptors and Müller glia seems to be initiated by Leukemia inhibitory factor (LIF) release from Müller glia^10^. Upregulation of LIF has been observed in a variety of retinal injury models^10–13^, and TNFα may be involved to induce LIF upregulation through the p38 MAPK pathway^14^. However, the precise signaling mechanism by which Müller glia sense retinal injury and activate injury responses is far from understood.

The aim of the present study was to characterize the molecular mechanisms regulating the proliferative and gliotic responses of Müller glia in the rat model of N-methyl-N-nitrosourea (MNU)-induced photoreceptor injury. We unexpectedly found that Müller glia play a predominant role in the phagocytic clearance of photoreceptor debris. We further demonstrated that phosphatidylserine (PS) and Rac1, key regulators of the phagocytic pathway, are involved to trigger the proliferative and gliotic responses of Müller glia after injury.

## Results

### Degenerated photoreceptors are eliminated in the absence of microglia/macrophage infiltration

**Figure 1:**
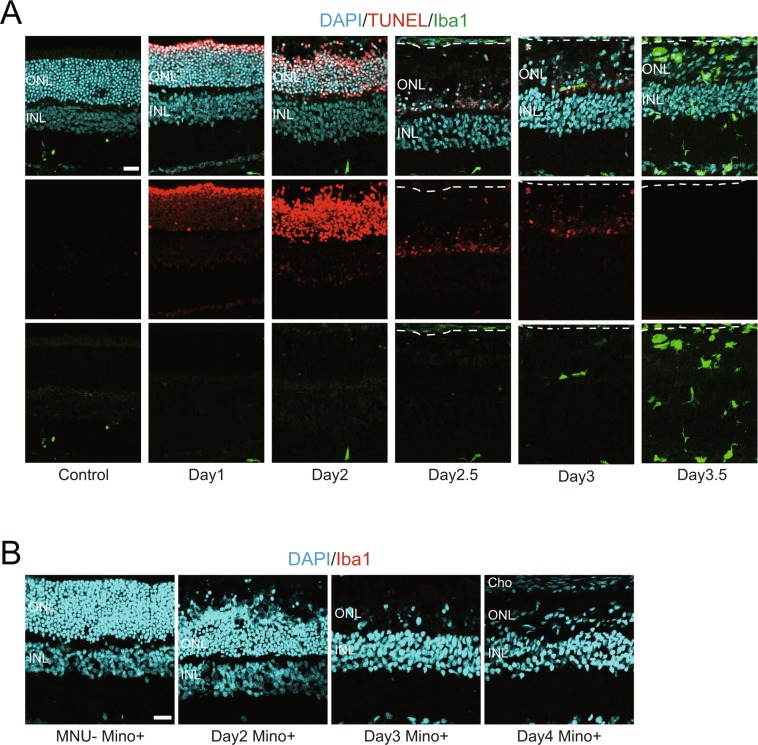
Elimination of degenerated photoreceptors in the absence of microglia/macrophage infiltration after MNU-induced retinal injury. (**A**) TUNEL assay (red), Iba1 immunohistochemistry (green), and nuclear counterstaining with 4′,6-diamidino-2phenyl-indole (DAPI, blue) showing photoreceptor-specific degeneration after MNU treatment and robust infiltration of activated microglia/macrophages into the ONL at day 3.5. Dotted lines indicate the border between the retina and the choroid. Note most TUNEL-positive photoreceptors are quickly eliminated by day 2.5. (**B**) Lack of infiltration of Iba1-positive microglia/macrophages after minocycline treatment (Mino+). Note most photoreceptors are eliminated by day 3. Cho: choroid, ONL: outer nuclear layer, INL: inner nuclear layer. Scale bar = 20 μm.

Previous studies have reported that degenerated photoreceptors are eliminated by professional phagocytes such as microglia and macrophages^15–20^. Therefore, we conducted immunofluorescence for Iba1, a microglia/macrophage marker, in combination with TdT-mediated dUTP nick end labeling (TUNEL) assay to assess whether degenerated photoreceptors were eliminated by microglia/macrophages (hereafter referred to collectively as macrophages) also in our retinal injury model. Most photoreceptor nuclei located in the outer nuclear layer (ONL) became TUNEL-positive by one day after MNU treatment (day 1), and this labeling was more intense at day 2 (Fig. 1A, Supplemental Fig. 1). Elimination of degenerated photoreceptors, especially those in the outer half of the ONL, was already evident at day 2, followed by almost complete loss of TUNEL-positive cells by day 2.5 (Fig. 1A). We expected to see infiltration of Iba1-labeled macrophages into the ONL between day 2 and 2.5, the timing at which marked elimination of degenerated photoreceptors occurred. Surprisingly, however, only a few macrophages were detected in the ONL between day 2 and 3, and robust infiltration of macrophages was observed only after day 3.5 (Fig. 1A). Whole-mount immunostaining also revealed a dramatic increase of Iba1-positive cells in the retina between day 3 and 4 (Supplemental Fig. 2A).

Above results suggest that macrophages may play only a limited role, if any, in the elimination of degenerated photoreceptors. To test this possibility, we inhibited the activation of macrophages by minocycline^21^ treatment after MNU-induced photoreceptor degeneration. Immunolabeling for Iba1 revealed significant inhibition of macrophage infiltration into the retina by minocycline treatment even at day 4, when numerous Iba1-positive macrophages were detected in the control retinas (Fig. 1B, Supplemental Fig. 2B). Inhibition of macrophage infiltration by minocycline was also confirmed using other macrophage markers including CD11β and CD68 (Supplemental Fig. 2C). Even after minocycline treatment, degenerated photoreceptors were mostly eliminated by day 3 (Fig. 1B). Together, these results suggest that macrophages may be dispensable for the elimination of degenerated photoreceptors in our rat injury model.

### Müller glia are involved in the clearance of photoreceptor debris

**Figure 2:**
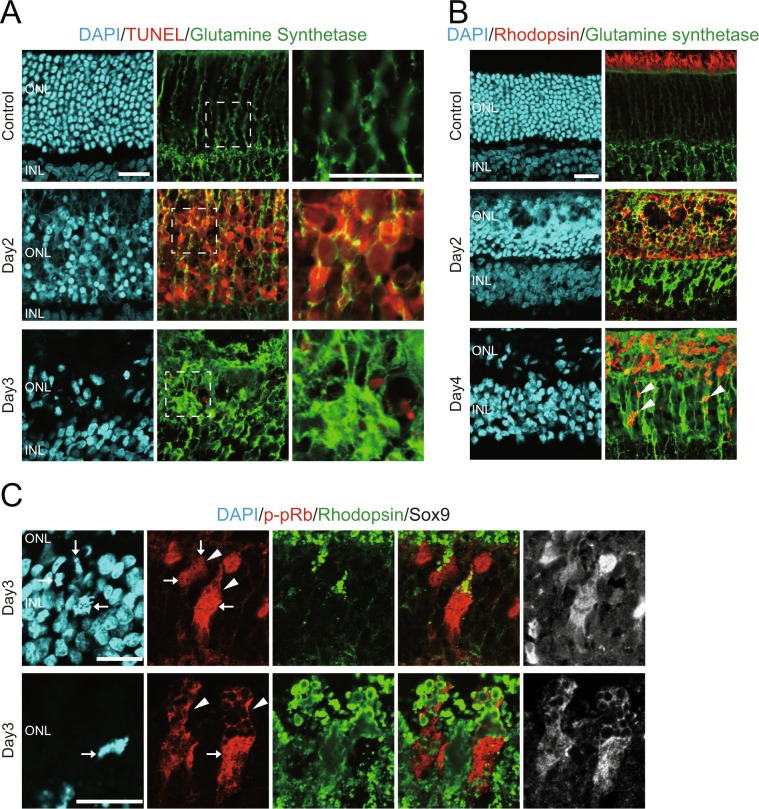
The presence of photoreceptor debris in the cytoplasm of Müller glia. (**A**) Immunofluorescence for glutamine synthetase (green) combined with TUNEL (red). Z-stack confocal images showing close association between photoreceptor debris and Müller glial processes. The areas marked with dotted squares are shown at higher magnification in the adjacent panels. (**B**) Double immunofluorescence for rhodopsin (red) and glutamine synthetase (green) showing rhodopsin-positive photoreceptor debris in the Müller glial processes. Arrowheads denote photoreceptor debris in the Müller glial processes located in the INL. (**C**) Triple immunofluorescence for phosphorylated-retinoblastoma protein (p-pRb, red), rhodopsin (green) and Sox9 (white). Note the presence of rhodopsin-positive photoreceptor debris within the cytoplasm of mitotic Müller glia labeled by p-pRb and Sox9. The condensed nuclei and p-pRb-positive cytoplasm of mitotic Müller glia are indicated by arrows and arrowheads, respectively. ONL: outer nuclear layer, INL: inner nuclear layer. Scale bar = 20 μm.

Given the previous reports that Müller glia possess phagocytic ability to remove degenerated cells^22–25^, we investigated whether Müller glia, rather than macrophages, phagocytosed degenerated photoreceptors in the MNU-treated rat retinas. We conducted immunofluorescence for glutamine synthetase (GS), a Müller glial cytoplasm marker^26^, in combination with TUNEL. Z-stack confocal images reconstructed from optical sections showed that TUNEL-positive photoreceptor nuclei were closely attached and surrounded by Müller glial processes at day2 (Fig. 2A). At day 3, some fragments of TUNEL-positive nuclei appeared to be within Müller glial processes; however, it was not clear whether those debris were indeed engulfed by Müller glia (Fig. 2A). We then conducted double immunofluorescence for GS and the photoreceptor marker rhodopsin and colocalization was examined on single optical sections. In the control retinas, rhodopsin signals were specifically detected in the photoreceptor outer segments while, in the MNU-treated retinas, a large number of rhodopsin-positive photoreceptor debris were observed in the ONL (Fig. 2B). Virtually all rhodopsin-positive debris were localized within GS-positive Müller glial processes (Fig. 2B). Notably, some rhodopsin-positive debris were observed in the Müller glial processes located in the inner nuclear layer (INL) away from the ONL, and such debris in the INL were most prominent at day 4 (Fig. 2B: arrowheads).

To further verify the presence of photoreceptor debris in Müller glia, we examined the localization of rhodopsin in Müller glia during mitosis. As reported previously^5^, most Müller glia reenter the cell cycle after MNU-induced photoreceptor injury, and Müller glia in the M phase was most frequently observed at day 3 after MNU treatment. Transcription-associated nuclear proteins are known to be displaced into the cytoplasm upon nuclear envelop breakdown during mitosis^27^. As we noted that both Sox9, a Müller glia-specific transcriptional factor, and phosphorylated-retinoblastoma protein (p-pRb), a cell cycle regulator, are dispersed in the cytoplasm of mitotic Müller glia (Supplemental Fig. 3), we performed triple immunofluorescence for Sox9, p-pRb, and rhodopsin to examine the presence of photoreceptor debris within the cytoplasm of mitotic Müller glia. As expected, rhodopsin-positive debris were colocalized with Sox9 and p-pRb in the cytoplasm of mitotic Müller glia located in the INL (Fig. 2C, upper panel), as well as in the ONL (Fig. 2C, lower panel) when examined at day 3. Taken together, these data strongly support the possibility that Müller glia phagocytose and clear degenerated photoreceptors prior to the infiltration of macrophages into the ONL.

### Phosphatidylserine recognition is required for the proliferation and phagocytic response of Müller glia after injury

**Figure 3:**
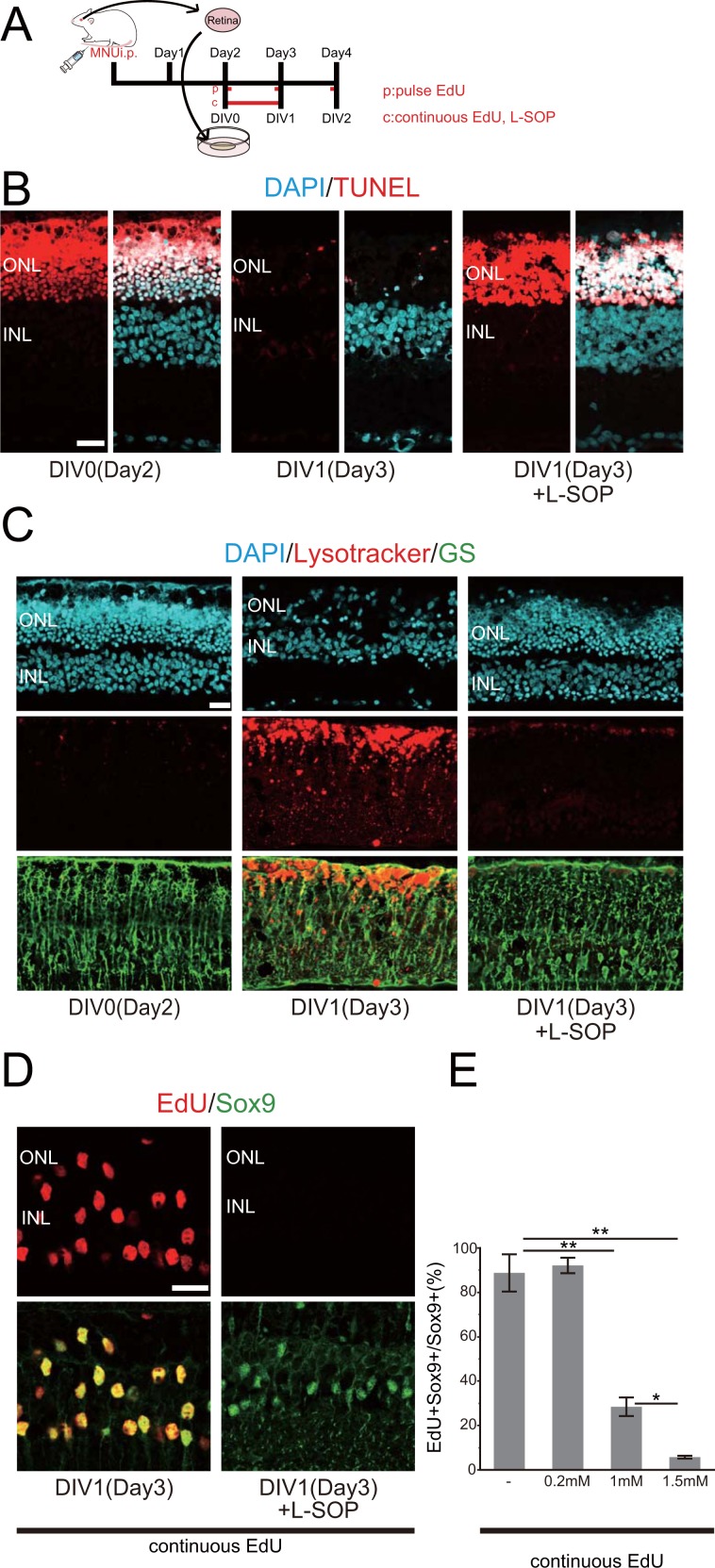
Inhibition of phosphatidylserine recognition prevents elimination of photoreceptor debris and proliferation of Müller glia. (**A**) The time schedule of explant cultures and the treatment with EdU and L-SOP. (**B**) TUNEL labeling (red) of the MNU-treated retinas explant-cultured with or without L-SOP. (**C**) Lysosome labeling with Lysotracker (red) combined with GS immunofluorescence (green) in the explants cultured with or without L-SOP. (**D**) 5-ethynyl-2′-deoxyuridine (EdU) incorporation assay (red) combined with Sox9 immunofluorescence (green) showing Müller glia proliferation in the explants cultured with or without L-SOP. (**E**) The proportions of EdU-positive Müller glia in the explants cultured under different concentrations of L-SOP. Each bar represents the mean ± standard error of the mean (SEM, n = 3). *P < 0.05, **P < 0.01. ONL: outer nuclear layer, INL: inner nuclear layer. Scale bar = 20 μm.

Above data indicate that most degenerated photoreceptors are phagocytosed by Müller glia between day 2 and 2.5. We have previously reported that most Müller glia reenter the G1 phase of the cell cycle by day 2, progressing into the S phase by day 2.5 in the MNU-treated rat retinas^5^. Thus, photoreceptor phagocytosis and cell cycle reentry seem to occur in parallel in Müller glia after photoreceptor injury. This leads to the hypothesis that the proliferation and phagocytic activity of Müller glia may be driven by a shared mechanism. To address this hypothesis, we used retinal explant culture, which allowed pharmacological manipulation of the retina and to exclude possible contributions of macrophages from outside the retina (Supplemental Fig. 4A). We first tested whether the phagocytic and proliferative responses of Müller glia *in vivo* can be replicated in the retinal explants. The rat retinas were excised and explanted at day 2 after MNU treatment (day 0 *in vitro*, DIV0) (Fig. 3A). Most TUNEL-positive degenerated photoreceptors were eliminated by DIV 1 (corresponding to day 3 *in vivo*), replicating the results *in vivo* (Fig. 3B). In addition, we found a drastic accumulation of lysosomes in Müller glia at DIV 1 using Lysotracker (Fig. 3C, Supplemental Fig. 4B), consistent with the enhanced phagocytic activity of Müller glia. We also confirmed the S phase entry of Müller glia in the explants by 5-Ethynyl-2′-deoxyuridine (EdU) incorporation assay. When the explants were pulse-labeled with EdU (Fig. 3A), the proportion of EdU-labeled Müller glia increased from 0 (DIV 0) to approximately 20% by DIV1, and subsequently decreased to approximately 1.4% by DIV 2 (Supplemental Fig. 4D). Under continuous presence of EdU (Fig. 3A), approximately 90% of Müller glia were labeled by DIV 1 (Fig. 3D,E). Taken together, these data confirmed that the phagocytic and proliferative responses of Müller glia observed *in vivo* were closely replicated in our retinal explant systems.

It is well established that phagocyte recognition of “eat me” signals, such as phosphatidylserine (PS), on apoptotic cells is required to initiate signaling events that lead to phagocytic engulfment^28–30^. We thus tested the possibility that both phagocytic and proliferative activities of Müller glia are regulated by PS recognition. To this end, we treated retinal explants with *O*-phospho-L-serine (L-SOP), a PS-related compound which blocks the PS-mediated signaling^31^. As expected, L-SOP treatment effectively inhibited the clearance of TUNEL-positive photoreceptors, as well as lysosome accumulation in Müller glia (Fig. 3B,C), indicating that PS recognition is required for phagocytic clearance of degenerated photoreceptors by Müller glia. Interestingly, cell cycle reentry of Müller glia, as assess by EdU incorporation, was also blocked almost completely by L-SOP treatment (Fig. 3D). The inhibitory effect of L-SOP on Müller glia proliferation was dose-dependent (Fig. 3E, Supplemental Fig. 4E). To exclude the possibility that L-SOP blocks Müller glia proliferation independently of its effect on PS recognition, we examined the effects of L-SOP on the proliferative activity of rMC-1, an immortalized rat Müller glial cell line. L-SOP treatment did not affect the proliferation and viability of rMC-1 cells (Supplemental Fig. 4F–H). These results suggest that PS recognition may be required for the activation of phagocytic and proliferative responses of Müller glia after photoreceptor injury.

### Rac1 activation is required for the phagocytic and proliferative responses of Müller glia after injury

**Figure 4:**
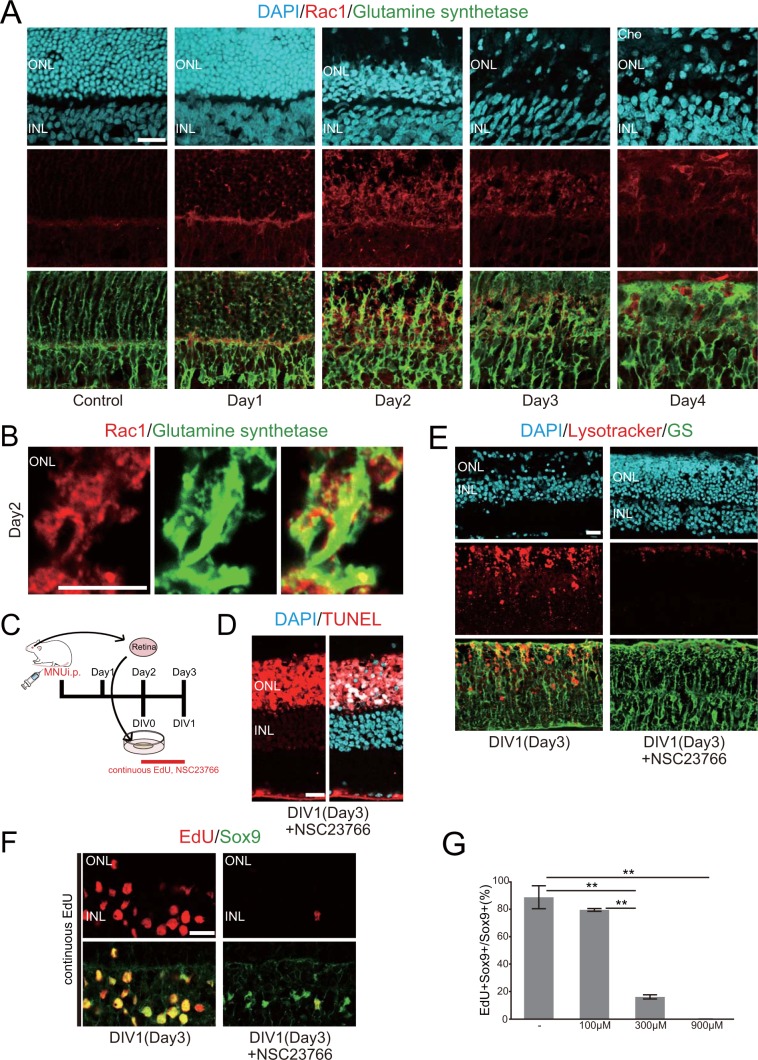
Inhibition of Rac1 activation prevents elimination of photoreceptor debris and proliferation of Müller glia. (**A**) Double immunofluorescence for Rac1 (red) and GS (green) in the MNU-treated retinas showing Rac1 accumulation in Müller glia. (**B**) High magnification images of Rac1 (red) and GS (green) at day 2 showing overall colocalization. (**C**) The time schedule of explant cultures and the treatment with EdU and the Rac1 inhibitor NSC23766. (**D**) TUNEL labeling (red) in the MNU-treated retinal explants cultured with NSC23766, showing the presence of photoreceptor debris at DIV1. (**E**) Lysotracker (red) combined with GS immunofluorescence (green) in the explants cultured with or without NSC23766, showing inhibition of lysosome accumulation by Rac1 inhibition. (**F**) EdU incorporation assay (red) combined with Sox9 immunofluorescence (green) in the explants cultured with or without NSC23766. Note significant inhibition of S phase entry of Müller glia by Rac1 inhibition. (**G**) The proportions of EdU-positive Müller glia in the explants cultured under different concentrations of NSC23766, showing dose-dependent inhibition of Müller glia proliferation. Each bar represents the mean ± SEM (n = 3). **P < 0.01. Cho: choroid, ONL: outer nuclear layer, INL: inner nuclear layer. Scale bars in A, D, E, F = 20 μm, in B = 10 μm.

PS recognition by phagocytes leads to the activation of the small GTPase Rac, which regulates cytoskeletal rearrangement and promotes the formation of phagocytic cup^32,33^. Therefore, we next tested whether Rac activation is involved in the phagocytic and proliferative responses of Müller glia in our retinal injury model. We first assessed the expression of Rac1 in the rat retina *in vivo* after MNU-induced photoreceptor injury by immunohistochemistry. Rac1 immunoreactivity was negligible in the control retinas, but it increased after MNU treatment with the peak intensity at day 2 (Fig. 4A). Between day 1 and day 3, Rac1 immunoreactivity was concentrated in the Müller glial processes located in the ONL, corresponding to the site of active phagocytosis of degenerated photoreceptors (Fig. 4A,B). At day 4, however, strong Rac1 staining was found in Iba1-positive macrophages, rather than in Müller glia (Fig. 4A, Supplemental Fig. 5A). We next examined the effects of Rac1 inhibitor NSC23766 on the phagocytic and proliferative activities of Müller glia in retinal explant cultures (Fig. 4C). Consistent with Rac1 expression in Müller glia, Rac1 inhibition blocked the elimination of degenerated photoreceptors (Fig. 4D) and lysosome accumulation in Müller glia (Fig. 4E). Furthermore, Rac1 inhibition dose-dependently blocked EdU incorporation by Müller glia (Fig. 4F,G, Supplemental Fig. 5B), indicating that Rac1 activation may be required for Müller glia proliferation after photoreceptor injury.

### Phosphatidylserine recognition and Rac1 activation are required for reactive gliosis of Müller glia

**Figure 5:**
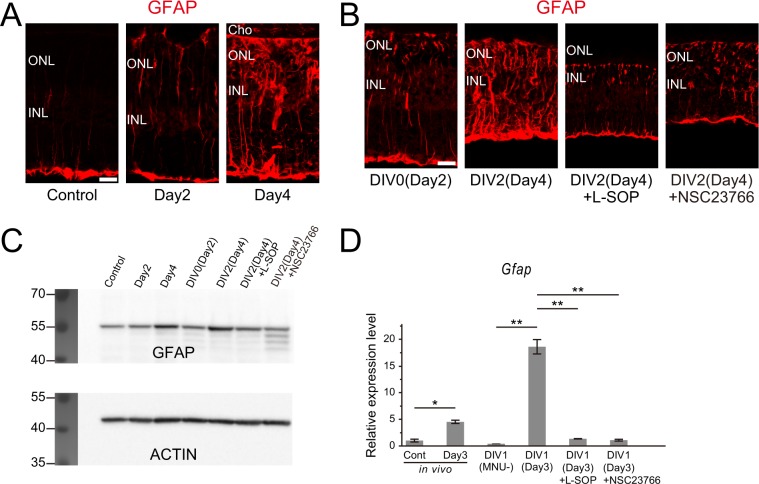
Inhibition of PS recognition and Rac1 activation prevents upregulation of glial fibrillary acidic protein (GFAP) expression in Müller glia. (**A**) Immunofluorescence for GFAP in the MNU-treated retinas showing significant GFAP upregulation by day 4. (**B**) Immunofluorescence for GFAP in the MNU-treated retinal explants cultured with L-SOP, NSC23766 or without inhibitors. GFAP upregulation is prevented by both inhibitors. Cho: choroid, ONL: outer nuclear layer, INL: inner nuclear layer. Scale bar = 20 μm. (**C**) Western blot analyses of GFAP using samples treated as (**A,B**). GFAP is upregulated by day 4 both *in vivo* and *in vitro*, and GFAP upregulation is inhibited by both L-SOP and NSC23766. Blots were cropped between 40 and 70 kDa for GFAP and 35 and 55 kDa for β-ACTIN. (**D**) Real-time PCR analyses of *Gfap* expression. *Gfap* mRNA levels are significantly increased after MNU treatment, but not in the presence of L-SOP and NSC23766. Each bar represents the mean ± standard deviation (SD) of three samples and the values expressed relative to controls (Cont) after normalization to *Gapdh* levels.

Reactive gliosis of Müller glia is characterized by cellular hypertrophy, migration, and upregulation of intermediate filaments such as GFAP^6^. Interestingly, inhibition of PS and Rac1 prevented not only the phagocytic activity and proliferation, but also cellular hypertrophy and migration of Müller glia after injury (Figs. 3D, 4F). To test the possibility that PS and Rac1 are involved to trigger injury-induced reactive gliosis of Müller glia, we next analyzed the effects of PS and Rac1 inhibition on GFAP expression in Müller glia. In the control retinas, intense GFAP immunolabeling was observed only in the nerve fiber layer, most likely reflecting the GFAP expression in astrocytes (Fig. 5A). GFAP expression was detected in some Müller glial processes by day 2 after MNU treatment and this staining was markedly increased by day 4 indicating reactive gliosis of Müller glia (Fig. 5A). Similar increase in GFAP immunolabeling was observed in the explanted retinas from DIV0 (day 2) to DIV2 (day 4) (Fig. 5B) whereas no such increase in GFAP was detected in the explants treated with L-SOP or NSC23766 (Fig. 5B). GFAP levels monitored by Western blotting verified immunofluorescence results (Fig. 5C, Supplemental Fig. 6). Upregulation of GFAP expression after injury and the effects of PS and Rac1 inhibition were also assessed by real-time RT-PCR analysis. GFAP mRNA levels were elevated after MNU treatment both *in vivo* and *in vitro* and the increase of GFAP expression was almost completely blocked by L-SOP or NSC23766 treatment (Fig. 5D). These data indicate that PS recognition and Rac1 activation are required to trigger reactive gliosis of Müller glia in our retinal injury model.

## Discussion

There is controversy as to which cell type or types are predominantly involved in the phagocytic removal of degenerated cells in the retina. While many studies have suggested the primary role of professional phagocytes such as microglia or bone marrow-derived macrophages^15–20^, other cell types such as Müller glia are also reported to show phagocytic activity during development^34,35^ or under pathological conditions^22–25^. A recent report has further suggested that Müller glia play a predominant phagocytic role during the early stage of photoreceptor degeneration in RhoP23H/P23H mice^36^. However, the extent to which Müller glia contribute to the clearance of cell debris in the retina and the mechanism regulating this function of Müller glia are poorly understood. The rat model of MNU-induced retinal injury is characterized by robust photoreceptor degeneration followed by immediate phagocytic response^22^. Here we present four lines of evidence indicating that Müller glia, rather than microglia/macrophages, are primarily responsible for removal of photoreceptor debris in this model. First, virtually all photoreceptor nuclei were quickly eliminated after MNU-induced damage prior to the infiltration of microglia/macrophages into the ONL. Second, inhibition of microglia/macrophage activation by minocycline treatment failed to affect phagocytic elimination of degenerated photoreceptors. Third, photoreceptor debris were effectively eliminated in the retinal explants which virtually lack microglia/macrophage. Finally, most rhodopsin-positive photoreceptor debris were found within the cytoplasm of Müller glia. Thus, our data are consistent with the notion that Müller glia may act as the primary phagocytes in the retina under some pathological condition. Phagocytic activity of Müller glia is commonly observed prior to overt infiltration of microglia/macrophages^24,25,36^. In the present model, infiltration of microglia/macrophages is observed only after day 3 of MNU treatment while, in other retinal injury models such as light damage^17^ or retinal detachment^19^, prominent infiltration and phagocytic activity of microglia/macrophages are observed within 24 hours of injury. Thus, the relative contribution of Müller glia in phagocytic clearance may depend, at least in part, on the timing of microglia/macrophage infiltration into the injured area. There also seem to be species differences in the phagocytic capacity of Müller glia. It has been reported that, in the MNU-treated mouse retinas, retinal pigment epithelial cells, but not Müller glia, cooperate with microglia/macrophages to remove photoreceptor debris^37,38^, which contrasts with the predominant phagocytic role of Müller glia in the rat MNU model. Further investigation is warranted to reveal how the phagocytic activity of retinal cells is regulated in diverse experimental models.

Retinal injury induces gliotic responses of Müller glia such as cellular hypertrophy, migration, proliferation, and GFAP upregulation^1^. Although a variety of secretory factors and intracellular signaling pathways have been implicated in the regulation of Müller glia proliferation and gliosis^6^, the precise signaling mechanism by which Müller glia sense retinal injury and activate the injury responses remains undefined. Here, we provide evidence that PS-mediated recognition of damaged photoreceptors by Müller glia initiates not only phagocytosis, but also proliferative and gliotic responses of Müller glia. This PS-mediated signaling may activate signal transduction pathways leading to upregulation of secretory factors, which may in turn trigger reactive responses of Müller glia. Alternatively, PS recognition may trigger proliferation and gliotic responses of Müller glia independently of secretory factors. A recent study using cell lines expressing MERTK and TIM4, well known engulfment receptors for apoptotic cells, has provided evidence that PS-mediated recognition of apoptotic cells can activate intracellular signaling pathways leading to proliferation of phagocytic cells^39^. It is thus likely that one or more types of phagocytic engulfment receptors expressed on Müller glia may be linked to distinct intracellular pathways activating a wide range of injury-induced responses of Müller glia.

Rac1, a member of the Rho family GTPases, is a critical regulator of a variety of cellular processes such as phagocytosis^40^. Rac1 acts downstream of phagocytic receptor activation and promotes the formation of phagocytic cups by regulating cytoskeletal rearrangement^32,33^. In the present study, Müller glia showed a robust increase in Rac1 expression after photoreceptor injury, and Rac1 inhibition was sufficient to prevent phagocytic clearance of degenerated photoreceptors by Müller glia. Furthermore, Rac1 inhibition blocked injury-induced proliferation and gliosis of Müller glia. These data indicate that Rac1 is essential not only for phagocytosis, but also for proliferative and gliotic responses of Müller glia. Our findings are consistent with the recent report that Rac1 regulates proliferation and gliosis of astrocytes after spinal cord injury^41^. There are also many studies indicating that Rac1 regulates cell cycle progression by upregulating cyclin D1, a key regulator of the G1/S transition^42,43^. As cyclin D1 expression is robustly increased in proliferating Müller glia after injury^5,44^, Rac1 may likely promote Müller glia proliferation by upregulating cyclin D1 expression. Accumulating evidence also indicates that Rac1 plays a key role in canonical Wnt signaling by promoting nuclear accumulation of β-catenin^45–47^. As Wnt signaling has been shown to promote Müller glia proliferation^48–50^, it is tempting to speculate that Rac1 may regulate Müller glia proliferation via its effects on Wnt signaling.

In conclusion, the present study revealed that injury responses of Müller glia including phagocytosis, proliferation, and GFAP upregulation are regulated in PS- and Rac1-dependent manners. PS and Rac1, key regulators of the phagocytic pathway, may play a role in crosstalk between different signaling pathways activated in Müller glia after injury. These data provide important insights into the mechanism by which glial cells coordinate their injury responses in the retina and in the central nervous system in general.

## Materials and Methods

### Animals and tissue preparation

Male Wistar rats (5 weeks old) obtained from Charles River Laboratories Japan (Yokohama, Japan) were maintained under a 12:12-hour light:dark cycle with food and water ad libitum. Animals were enucleated under euthanasia with isoflurane. All animal experiments were designed according to the institutional ethical code for laboratory animal and were approved by the institutional review committee (Tokyo Women’s Medical University, Approve No. AE19-051).

Tissue preparation for immunohistochemistry was performed as described previously^5^. Briefly, the eyecups without lens were fixed with 4% paraformaldehyde in phosphate buffered saline (PBS) for 1 hour on ice, frozen in OCT compound (Leica Biosystems, Nussloch, Germany) with dry ice-isopentane, and cut on a cryostat into serial vertical sections of 10 μm through the optic disc along the dorsoventral axis.

### Induction of retinal degeneration

To induce photoreceptor degeneration, N-methyl-N–nitrosourea (MNU, Sigma-Aldrich, St. Louis, MO, USA) was intraperitoneally injected into rats at the dose of 70 mg/kg body weight as described previously^5^.

### Minocycline treatment

Minocycline (Tokyo Chemical Industry, Tokyo, Japan) was intraperitoneally injected into rats at the dose of 50 mg/kg twice a day at intervals of 12 hours, beginning at 1 day before MNU treatment until sacrificed.

### Retinal explant

Rat retinas were excised and explanted 2 days after MNU injection. The retinas were isolated in sterilized PBS, spread on membranes placed in 6-well plates and incubated in 1 ml of retinal medium^51^ containing Minimum Essential Medium (MEM)–Hepes (Invitrogen,San Diego, CA) supplemented with 25% Hanks balanced salt solution (Invitrogen), 25% heat-inactivated horse serum, glucose (final concentration, 5.75 mg/ml), 200 μM L-glutamine and 50 units/ml penicillin-streptomycin in a humidified atmosphere of 5% CO_2_ in air at 37 °C. To visualize lysosomes, Lysotracker (Thermo Fisher Scientific, Waltham, MA USA) was added to the medium at the dose of 1 μl/ml. A phosphatidylserine mimic, O-Phospho-L-serine (L-SOP), was directly dissolved in the medium at the concentrations of 0.2 mM, 1 mM or 1.5 mM. Rac1 inhibitor NSC23766 (Merck, Darmstadt, Germany) dissolved in sterilized water was added to the medium at the final concentrations of 100 μM, 300 μM or 900 μM. Frozen sections of retinal explants were prepared as described above.

### Cell culture and treatments

Rat-derived Müller glial cell line, rMC-1, was obtained from Applied Biological Materials (Vancouver, Canada). Cells were seeded on poly-L-lysine-coated culture cover glasses (Matsunami Glass, Osaka, Japan) in 24-well culture dishes and grown in Dulbecco’s Modified Eagle Medium (D-MEM) high glucose supplemented with 10% fetal bovine serum (FBS), 50 units/ml penicillin-streptomycin in a humidified atmosphere of 5% CO_2_ in air at 37 °C. An appropriate number of exponentially growing rMC-1 was seeded 1 day before treatment with L-SOP. L-SOP was directly added to the cell culture at the concentration of 1.5 mM, and cells were incubated for 24 hours until fixed by 4% paraformaldehyde in PBS for 15 minutes at room temperature.

### EdU incorporation assay

5-ethynyl-2′-deoxyuridine (EdU) incorporation assay for explanted retinas and rMC-1 cells were performed with use of Click-iT™ Plus EdU Alexa Fluor™ 555 Imaging Kit (Thermo Fisher Scientific, Waltham, MA USA) according to the manufacturer’s instructions. For EdU pulse labeling, explanted retinas were treated with EdU for 2 hours before fixation. Control explants (DIV0) were cultured in the EdU-containing medium for 2 hours from immediately after collection until fixation. For continuous labeling, explants were cultured under the sustained presence of EdU. The rMC-1 cells were pulse-labeled with EdU for 2 hours before fixation.

### Immunofluorescence

Immunofluorescence staining was performed as described previously^5^. Primary and secondary antibodies are listed in Supplementary Table S1. Cell nuclei were counterstaining with 4′, 6-diamidino-2phenyl-indole 384 (DAPI; Sigma Aldrich). Immunofluorescence images were collected using a confocal laser scanning microscope (LSM710; Carl Zeiss, Jena, Germany).

### TUNEL assay

Cell death was visualized by terminal deoxynucleotidyl transferase-mediated dUTP nick end-labeling (TUNEL) assay using *in situ* cell death detection kit, TMR red (Roche, Mannheim, Germany), as described previously^5^.

### Cell counting

Cell counting were conducted on vertically sliced retina as described previously^5^. Two fields per section (four sections per animal, three animals per stage) were analyzed under a fluorescence microscope (Eclipse E600, Nikon Instruments, Tokyo, Japan), excluding the periphery of the tissue. To count culture cells, the automatic counting tool of ImageJ (National Institutes of Health, Bethesda, MD, USA) was used.

### Western blotting

Western blotting was carried out as described previously^5^. Briefly, tissues were lysed with RIPA buffer supplement with protease inhibitor (Nacalai Tesque, Kyoto, Japan). The lysates were resolved by SDS-PAGE and immunoblotting was performed using anti-GFAP antibody (Sigma Aldrich) and anti-β-actin antibody (Sigma Aldrich). The immunoblots were visualized with HRP-conjugated donkey anti-mouse IgG (Jackson Immuno Research, West Grove, PA, USA) using enhanced chemiluminescence reagent Immunostar (WAKO, Osaka, Japan).

### Quantitative (real-time) RT-PCR

Total RNA was extracted from the retinas collected from one eye of three animals using the RNeasy plus kit (QIAGEN, Hilden, Germany). RNA was reverse transcribed using the ReverTra Ace qPCR RT Master Mix with gDNA Remover Kit (Toyobo, Osaka, Japan). Quantitative PCR was carried out by Step One Plus and Power SYBR Green PCR Master Mix (Thermo Fisher Scientific). Primers used are shown in Supplementary Table S2. Data were normalized to *Gapdh* expression and the means of three samples were presented relative to control level.

### Statistical analysis

Statistical analysis was conducted by the Student’s t-test or the one-way analysis of variance (ANOVA) followed by Tukey-Kramer honest significant difference (HSD) using the JMP software (SAS Institute, Cary, NC, USA). A value of P < 0.05 was considered to be statistically significant.

## Data Availability

The datasets generated during and/or analyzed during the current study are available from the corresponding author on reasonable request.

## Supporting Information

Supporting Information.
