# Supplementary material for: Phosphatidylserine recognition and Rac1 activation are required for Müller glia proliferation, gliosis and phagocytosis after retinal injury
Source: Sci Rep. 2020 Jan 30;10:1488. doi: 10.1038/s41598-020-58424-6 (PMC6992786; doi:10.1038/s41598-020-58424-6)
Supplement: Supplementary file 1 — Supporting Information. [file 41598_2020_58424_MOESM1_ESM.pdf]

Manuscript Title:

Phosphatidylserine recognition and Rac1 activation are required for Müller glia proliferation, gliosis and phagocytosis after retinal injury.

Author names:

Kaori Nomura-Komoike, Fuminori Saitoh, and Hiroki Fujieda

Supplementary Figure 1: TUNEL signals in the MNU-treated retinas. Confocal images with parameter settings optimized for the signals at day 1 (middle panel) and day 2 (right panel). ONL: outer nuclear layer, INL: inner nuclear layer. Scale bar=20μm

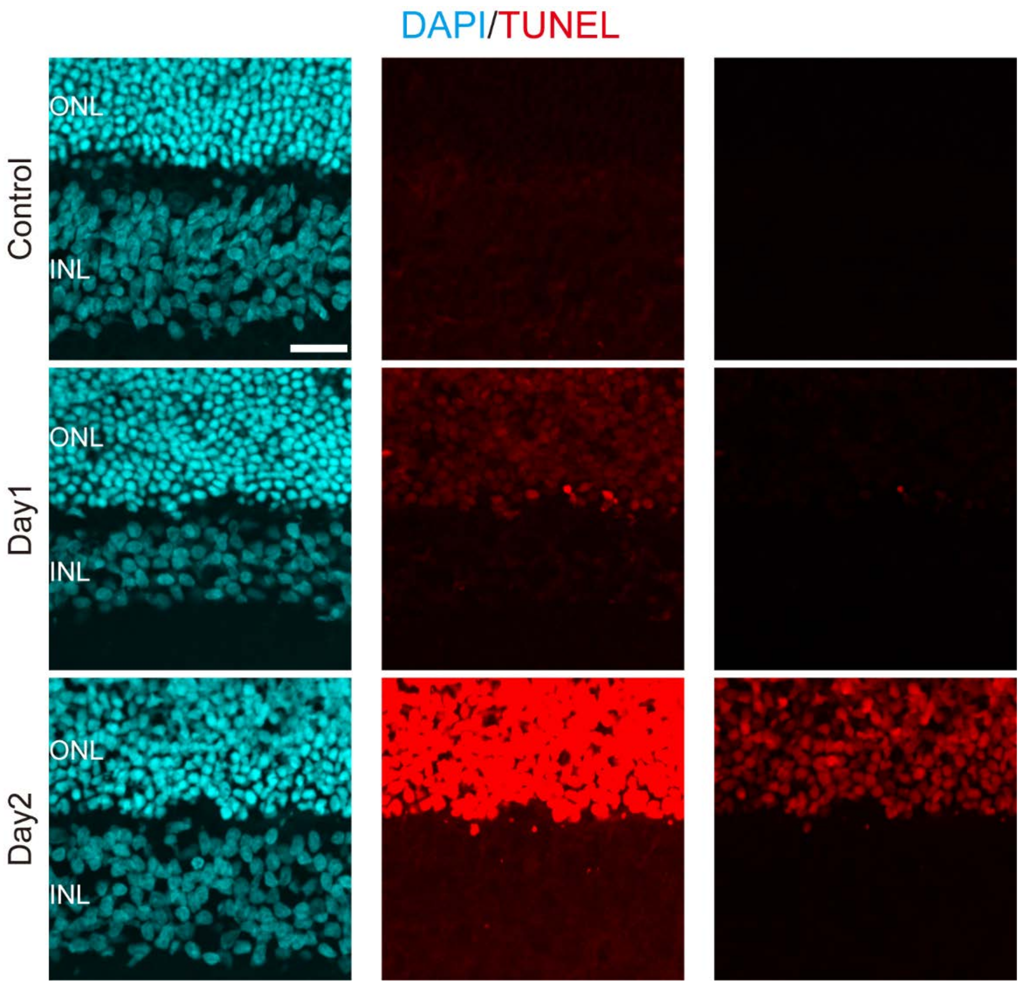

Supplementary Figure 2: Minocycline inhibits microglia/macrophage infiltration into the retina after MNU-induced injury. (A, B) Immunofluorescence for Iba1 using the whole mount retinas. Horizontal optical sections at the level of the ONL. (C) Double immunofluorescence for macrophage markers (CD11β /Iba1 or CD68/Iba1) using vertical retinal sections. Cho: choroid, ONL: outer nuclear layer, INL: inner nuclear layer, Mino: Minocycline. Scale bar= 20μm.

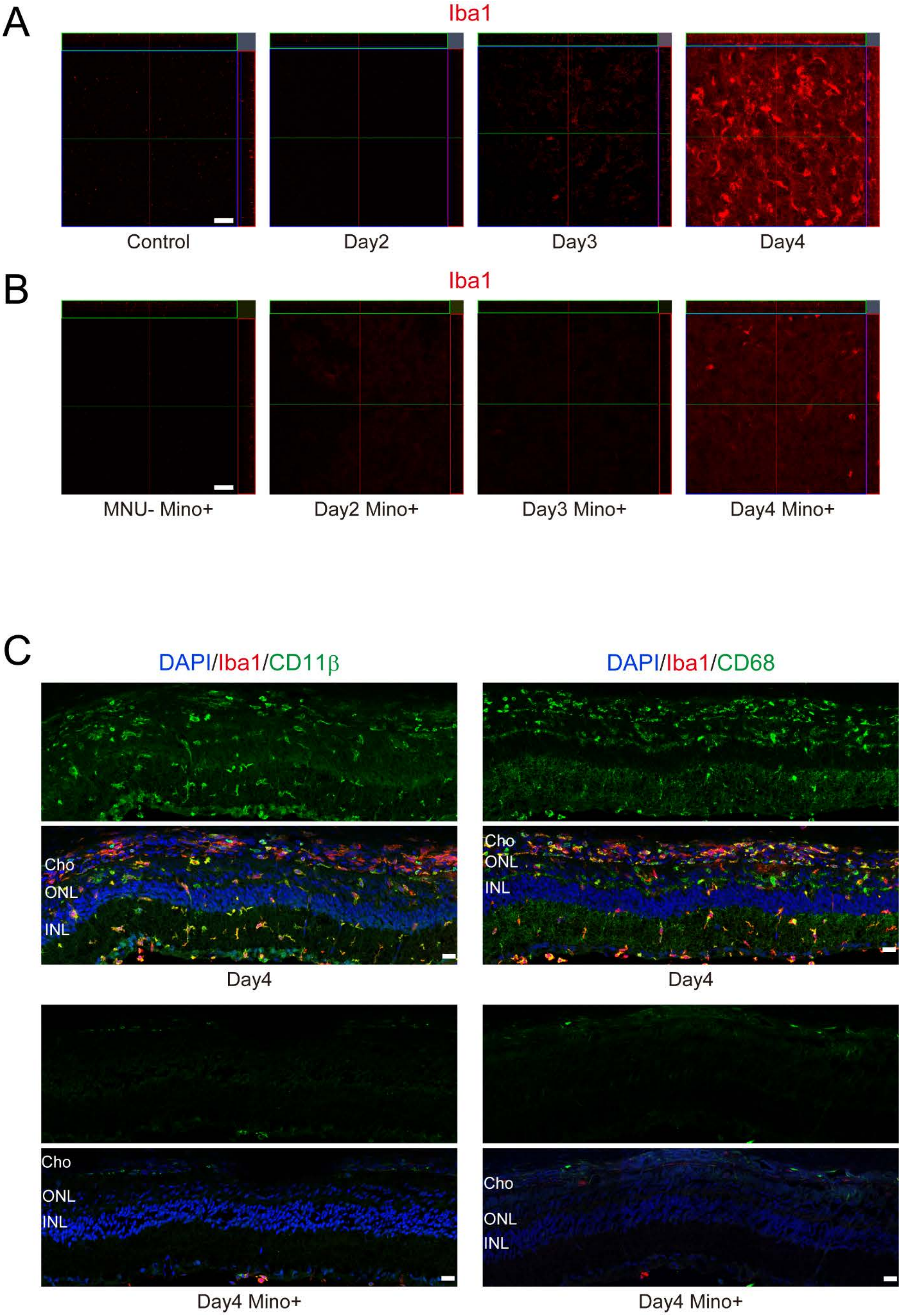

Supplementary Figure 3: Localization of Sox9 and phosphorylated-retinoblastoma protein (p-pRb) in the cytoplasm of mitotic Müller glia. (A) Triple immunofluorescence for phospho-histone H3 (pH3, M-phase marker), S100β (Müller glia cytoplasmic marker), and Sox9 (Müller glia nuclear marker). Note the presence of Sox9 labeling in the cytoplasm of mitotic Müller glia (arrows). (B) Triple immunofluorescence for pH3, p-pRb, and Sox9. Showing colocalization of p-pRb and Sox9 in the cytoplasm of M-phase Müller glia (arrows). ONL: outer nuclear layer, INL: inner nuclear layer. Scale bar= 20μm.

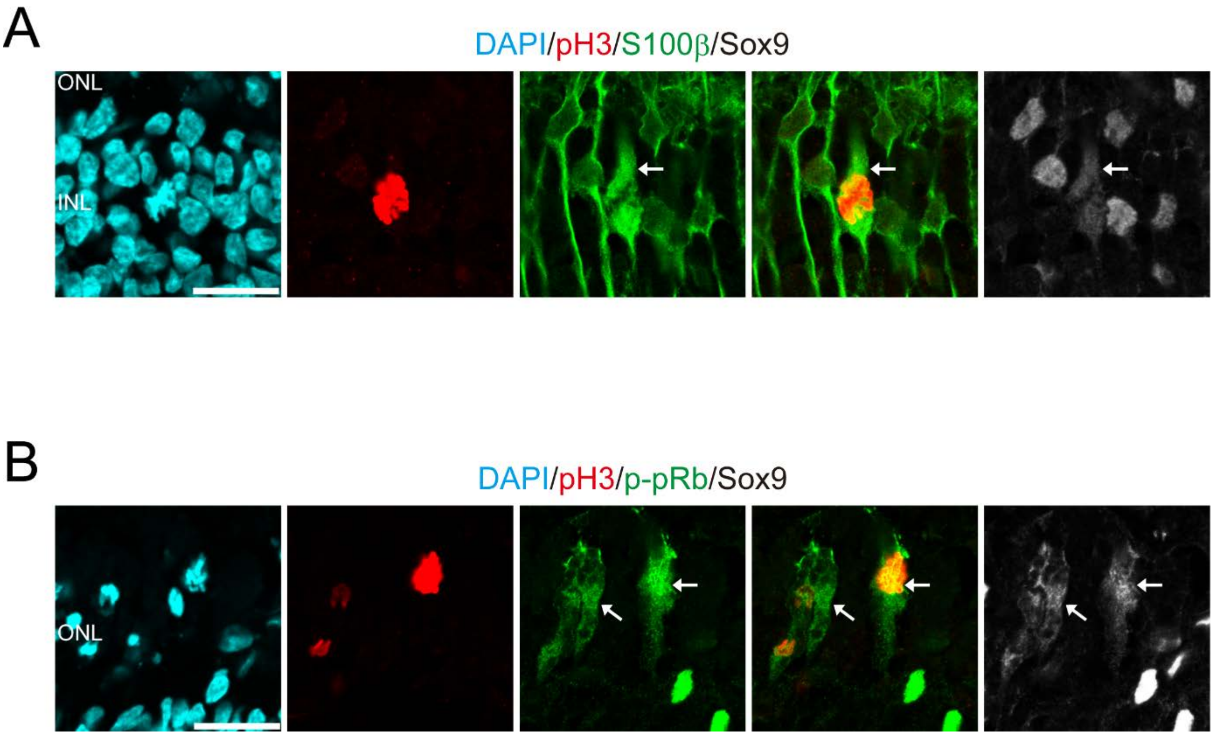

Supplementary Figure 4: Characterization of retinal explants and the effects of phosphatidylserine (PS) inhibition by *O*-phospho-L-serine (L-SOP). (A) Immunofluorescence for Iba1 in the explanted retinas showing the absence of microglia/macrophages. (B) Lysosomes stained with Lysotracker and a mitotic Müller glial cell identified by cytoplasmic p-pRb immunofluorescence. (C) EdU-pulse labeling with Sox9 immunofluorescence in the explanted retinas showing Müller glia in S phase. (D) Quantification of EdU-positive Müller glia. Bars represent the mean ± standard error of the mean (SEM, n=3). ***P*<0.01. (E) Continuous EdU labeling with Sox9 immunofluorescence in the explanted retinas treated with different concentrations of L-SOP. (F) EdU-pulse labeling in rMC-1 cells with/without L-SOP treatment. (G) Quantification of EdU-positive rMC-1 cells. Bars represent the mean ± SEM (n=3). (H) Quantification of DAPI-labeled rMC-1 cells. Bars represent the mean ± SEM (n=3). ONL: outer nuclear layer, INL: inner nuclear layer. Scale bar= 20μm.

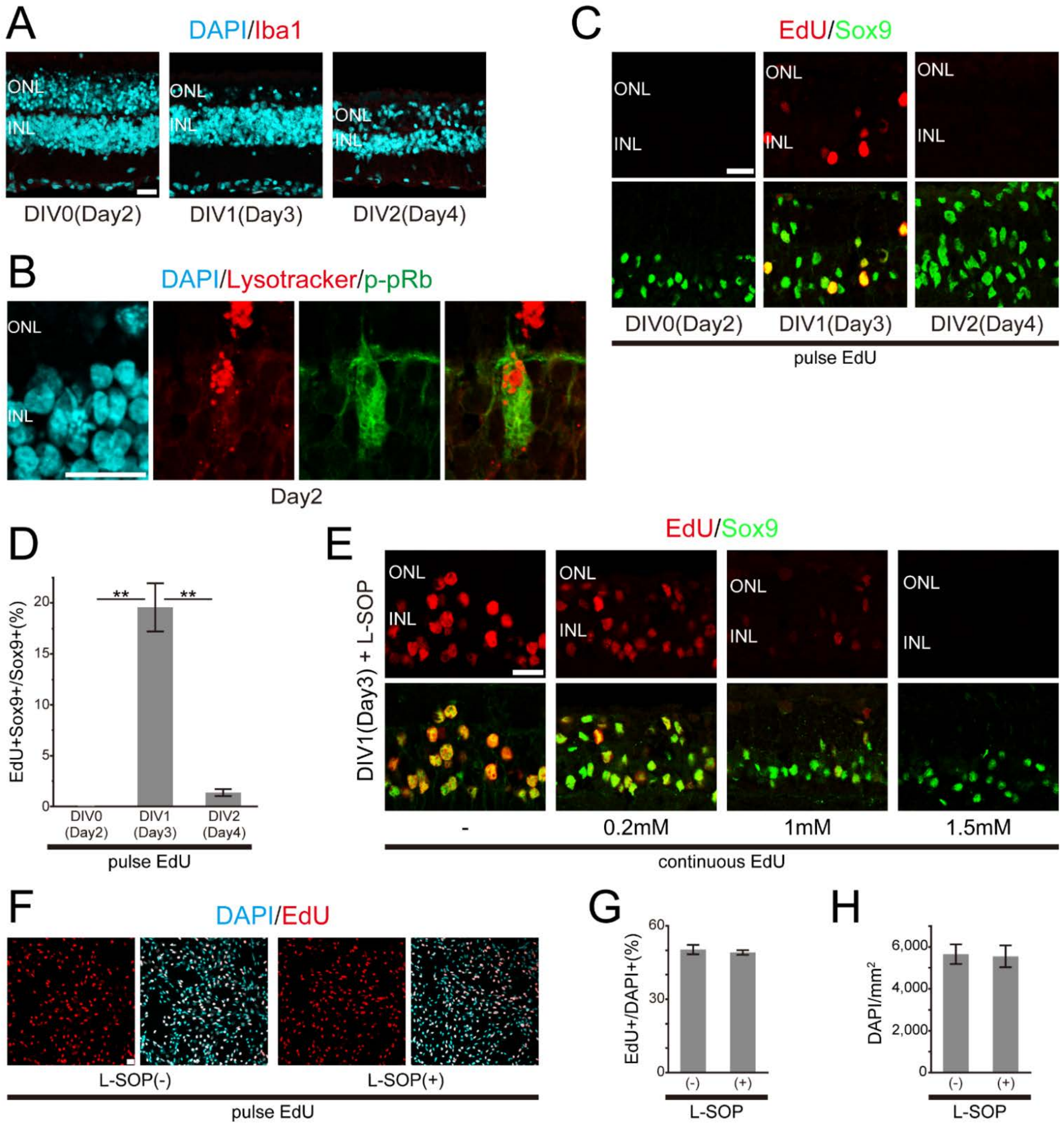

Supplementary Figure 5: The effects of Rac1 inhibition on Müller glia proliferation. (A) Expression of Rac1 in Iba1-positive microglia/macrophages at day 4. (B) Continuous EdU labeling with Sox9 immunofluorescence in the explanted retinas treated with different concentrations of Rac1 inhibitor NSC23766. ONL: outer nuclear layer, INL: inner nuclear layer. Scale bar= 20μm.

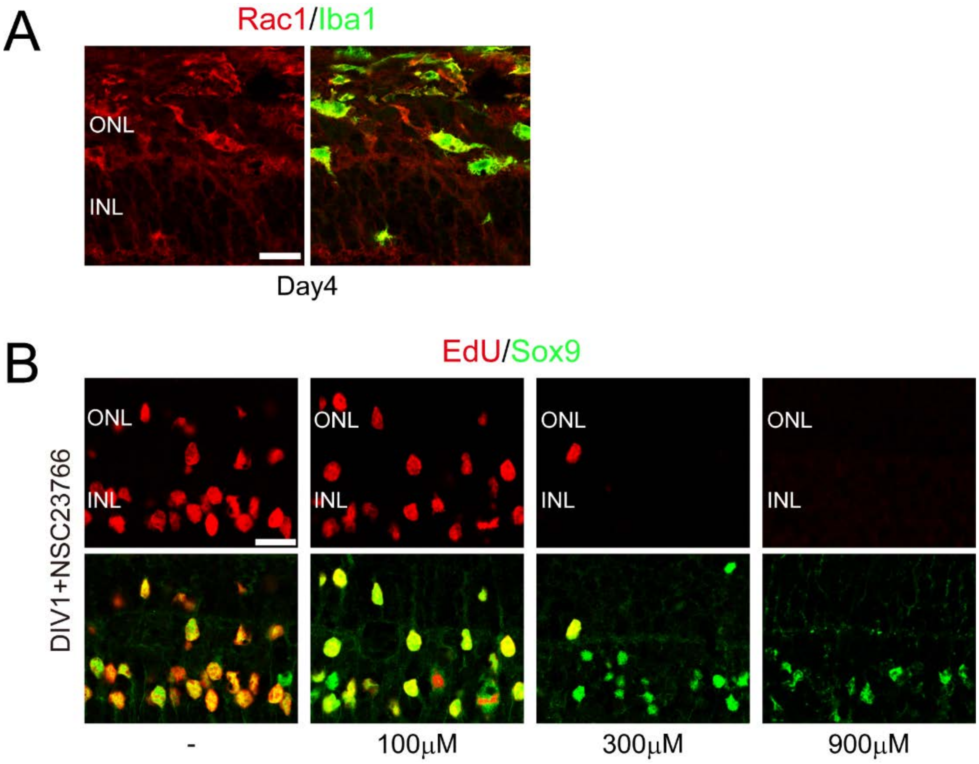

Supplementary Figure 6: Full length blots of GFAP and β-Actin.

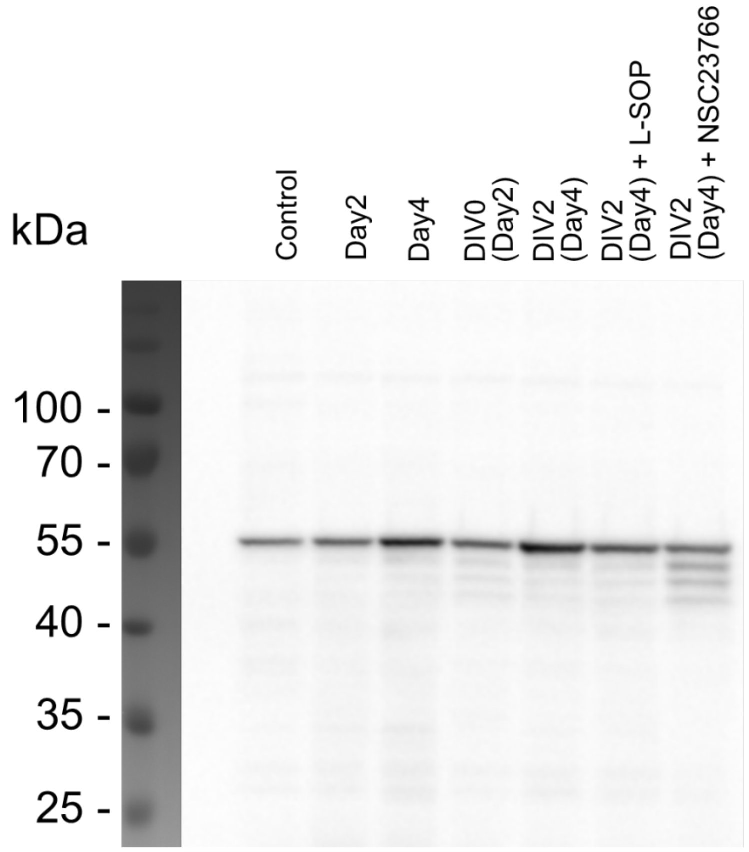

GFAP

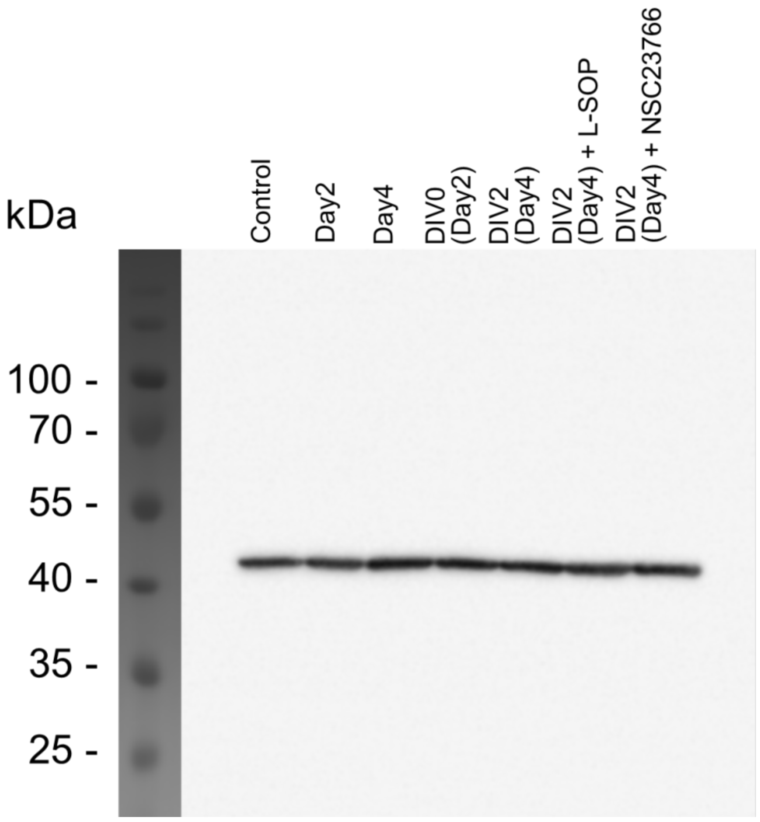

ACTIN

Supplementary Table S1

Primary antibodies

Antibody	Dilution	Species	Source
Iba1	2000	Rabbit	Wako 019-19741
CD11β	500	Mouse	serotec MCA275R
CD68	100	Mouse	serotec MCA341R
Glutamine synthetase	5000	Rabbit	Sigma G2781
Rhodopsin	5000	Mouse	Sigma O4886
Phospho-pRb	1000	Rabbit	Cell Signaling 9308
Sox9	2000	Rabbit	Millipore AB5535
Sox9	2000	Goat	R&D Systems AF3075
Phospho-histion H3	1000	Rabbit	Upstate 06-570
Phospho-histion H3	10000	Mouse	Upstate 05-806
S100β	2000	Mouse	Sigma S2532
Rac1	2000	Mouse	BD 610650
GFAP	1000	Rabbit	Cosmobio SML-ROI003

Secondary antibodies

Antibody	Dilution	Source
donkey anti-rabbit IgG Alexa Fluor 555	1000	Invitrogen A-31572
donkey anti-rabbit IgG Alexa Fluor 488	1000	Invitrogen A-21206
donkey anti-mouse IgG Alexa Fluor 555	1000	Invitrogen A-31570
donkey anti-mouse IgG Alexa Fluor 488	1000	Invitrogen A-21202
donkey anti-goat IgG Alexa Fluor 647	1000	Invitrogen A-21447

Supplementary Table S2

Primers

Gene	Forward	Reverse
Gapdh	ACAAGATGGTGAAGGTCGGTGTGA	AGCTTCCCATTCTCAGCCTTGACT
Gfap	CGGAGACGTATCACCTCTG	AGGGAGTGGAGGCGTCATTCG
